# Precision nutrition in critical illness: a phase-specific framework from acute feeding to post-ICU recovery

**DOI:** 10.3389/fnut.2026.1859625

**Published:** 2026-07-10

**Authors:** Pengcheng Tian, Ming Fan, Chaolin Huang

**Affiliations:** 1 Wuhan Jinyintan Hospital, Tongji Medical College of Huazhong University of Science and Technology, Wuhan, China; 2 Hubei Clinical Research Center for Infectious Diseases, Wuhan, China; 3 Wuhan Research Center for Communicable Disease Diagnosis and Treatment, Chinese Academy of Medical Sciences, Wuhan, China; 4 Joint Laboratory of Infectious Diseases and Health, Wuhan Institute of Virology and Wuhan Jinyintan Hospital, Chinese Academy of Sciences, Wuhan, China; 5 Institute of Hematology, Union Hospital, Tongji Medical College, Huazhong University of Science and Technology, Wuhan, China

**Keywords:** critical illness, energy target, enteral nutrition, feeding intolerance, parenteral nutrition, post-intensive care recovery, precision nutrition, protein target

## Abstract

Precision nutrition in critical illness should be understood as a longitudinal clinical strategy rather than a fixed prescription for calorie and protein delivery. This structured narrative review used PubMed/MEDLINE as the primary database and supplemented the search through Web of Science Core Collection and Google Scholar. Publications from January 2020 to January 2026 were prioritized, while earlier landmark trials, guidelines, and consensus papers were retained when relevant. Evidence from 73 references, including guidelines, randomized trials, systematic reviews, and relevant observational or mechanistic studies, was synthesized. We propose an operational framework with bedside indicators for early acute illness, stabilization/prolonged ICU care, and post-ICU recovery. During early acute instability, safe initiation and tolerance-based progression are favored over immediate completion of calculated targets. During stabilization and recovery, greater emphasis is placed on correcting persistent deficits, preserving lean mass, and supporting rehabilitation. Feeding intolerance is interpreted as gastrointestinal dysfunction, avoidable interruption, aspiration risk, or persistent inability to sustain enteral nutrition. Post-pyloric feeding and supplemental parenteral nutrition are selective escalation strategies, with safeguards against overfeeding and metabolic complications. After ICU discharge, quantified intake, swallowing function, muscle mass, functional trajectory, and participation in rehabilitation should guide continued support. The framework contextualizes existing recommendations and requires prospective validation.

## 1 Introduction

Nutritional support in critical illness has traditionally been framed around several practical questions: when to start feeding, which route to use, and how much energy and protein to deliver (1). These questions remain essential, but they are insufficient when applied without the physiological context of illness. Critical illness is not a homogeneous metabolic state (2–4). Patients admitted to the intensive care unit differ substantially in diagnosis, inflammatory burden, organ dysfunction, body composition, nutritional reserve, gastrointestinal function, exposure to sedation and vasopressors, and expected duration of critical care. Even within the same individual, nutritional priorities change over time as the disease evolves from acute organ failure to stabilization and then to prolonged recovery (5–7).

This heterogeneity limits the usefulness of a static, one-size-fits-all prescription (8, 9). Current guidelines from ESPEN (3, 4), ASPEN/SCCM (10, 11), and other expert groups provide an essential framework by emphasizing early nutritional intervention, preference for enteral nutrition when feasible, attention to protein provision, and the use of indirect calorimetry when available. However, bedside decisions remain difficult because guideline targets are often interpreted as static values, actual delivery is reduced by intolerance and interruptions, and nutritional vulnerability frequently persists after ICU discharge (7, 9, 12–20).

Previous expert recommendations and narrative reviews have already argued for personalized critical care nutrition (1, 2, 8). The unresolved problem is implementation. As a result, protein and energy targets are frequently discussed without sufficient reference to disease phase, prescribed goals are often conflated with actual delivery, and nutritional vulnerability after ICU discharge remains underintegrated into the overall nutrition strategy. We argue that these are not separate problems, but expressions of a common conceptual limitation: nutritional adequacy in critical illness has been treated as a fixed numeric objective rather than a phase-dependent and delivery-constrained clinical process.

We integrate three problems that are often discussed separately: physiological phase, the gap between prescribed and delivered nutrition, and the transition from ICU survival to functional recovery. The resulting framework provides explicit bedside indicators for phase assignment and practical escalation points for indirect calorimetry, post-pyloric feeding, and supplemental parenteral nutrition (PN). It is intended to contextualize existing recommendations, not replace them or claim validation as a clinical algorithm.

## 2 Methods

The literature search was designed to identify key evidence relevant to the development of a phase-specific precision nutrition framework for adult critically ill patients. PubMed/MEDLINE was used as the primary biomedical database. Supplementary identification was performed through Web of Science Core Collection and Google Scholar. The search focused on publications from January 2020 to January 2026, while earlier landmark trials, guidelines, and consensus papers published between 2011 and 2019 were retained when they were essential to the interpretation of current evidence.

The core PubMed syntax was: ((“critical illness”[Title/Abstract] OR “critically ill”[Title/Abstract] OR “ICU”[Title/Abstract] OR “intensive care”[Title/Abstract]) AND (“precision nutrition”[Title/Abstract] OR “personalized nutrition”[Title/Abstract] OR “phase-specific”[Title/Abstract] OR “protein target”[Title/Abstract] OR “energy debt”[Title/Abstract] OR “feeding intolerance”[Title/Abstract] OR “post-ICU”[Title/Abstract] OR rehabilitation[Title/Abstract])) AND (Review[Publication Type] OR Meta-Analysis[Publication Type] OR Randomized Controlled Trial[Publication Type] OR Guideline[Publication Type]). The equivalent Web of Science topic search used TS = (“critical illness” OR “intensive care”) AND TS = (“precision nutrition” OR “protein target” OR “energy target” OR “enteral nutrition” OR “parenteral nutrition” OR “feeding intolerance” OR “indirect calorimetry” OR “post-ICU recovery” OR rehabilitation). Reference lists of major guidelines, reviews, and landmark trials were also examined.

Using this predefined search string, the initial PubMed/MEDLINE search yielded 821 records. Eligible evidence addressed adults with critical illness and at least one predefined domain: phase-specific protein or energy delivery, EN feasibility, feeding intolerance, supplemental PN, indirect calorimetry, or post-ICU nutritional recovery. International guidelines, randomized controlled trials (RCTs), systematic reviews/meta-analyses, and influential observational or mechanistic studies were prioritized. Pediatric-only reports, studies outside critical care, duplicate reports without additional relevant findings, conference abstracts, and publications without direct relevance to the predefined domains were excluded. Candidate records were assessed for conceptual relevance to the framework; 73 sources were retained in the cited synthesis. Screening was not performed independently in duplicate, and no PRISMA flow diagram was generated.

## 3 Why illness phase should guide nutritional interpretation

### 3.1 Metabolic heterogeneity across acute, stabilization, and recovery phases

Critical illness is metabolically dynamic, not static. Wernerman et al. (5) described changing inflammation, catabolism, endogenous substrate mobilization, and organ-support needs over time, while Reignier et al. (6) emphasized that early nutrition must account for organ failure, hemodynamic instability, and feeding tolerance. These sources support phase-specific nutrition, but do not define universally validated phase boundaries. Stabilization is characterized by improving perfusion, stable or decreasing organ support, greater route feasibility, and increasing relevance of cumulative deficits. Recovery begins when acute organ failure is resolving and the main goals shift toward oral intake, mobilization, tissue rebuilding, and functional independence (7, 16).

In this review, phases are therefore identified physiologically rather than by ICU day alone. The early acute phase is suggested by unresolved shock, unstable perfusion, substantial vasoactive or organ support, and unreliable gastrointestinal tolerance. Stabilization is suggested by improving perfusion, stable or decreasing support, absence of new organ failure, and increasing feasibility of EN advancement. Recovery begins when organ failure is resolving and goals shift toward oral intake, mobilization, tissue rebuilding, and functional independence (5–7, 16). Van Zanten et al. (7) and Singer (16) support extending nutrition care into post-ICU recovery, but standardized recovery-phase criteria remain incomplete. Phase assignment should therefore be dynamic, because patients may move backward if shock, organ-support needs, or feeding intolerance recur.

### 3.2 Why the same nutritional intake may have different meanings across phases

The practical importance of disease phase lies in the changing purpose of nutritional therapy. In the early acute phase, ESPEN guidelines by Singer et al. (3, 4) emphasize progressive energy delivery and avoidance of overfeeding, while ASPEN/SCCM guidance by Compher et al. (10) and McClave et al. (11) supports early enteral nutrition when feasible but stresses that delivery should depend on hemodynamic stability and gastrointestinal tolerance. Wischmeyer et al. (1) further recommend cautious early energy and protein advancement, particularly in patients with shock, high vasopressor exposure, acute kidney injury, or high illness severity. Patel et al. (12) also supported early use of the gut by emphasizing the contemporary rationale for enteral nutrition in critically ill adults, but this rationale is best interpreted as support for feasible and safe enteral initiation rather than for aggressive correction of all calculated deficits. This interpretation is consistent with the PermiT trial by Arabi et al. (21) and the trophic-feeding trial by Rice et al. (22), which did not show clear superiority of early full caloric replacement in selected critically ill patients.

However, these early-phase feeding trials should not be extrapolated to prolonged or chronic critical illness. Castro et al. specifically examined high versus low protein intake in chronic critical illness, highlighting that protein exposure may have different relevance once acute instability gives way to prolonged catabolism and recovery-oriented care (23). Wernerman et al. (5) and Reignier et al. (6) described critical illness as a dynamic metabolic trajectory from acute organ failure and catabolism toward recovery, progressive intake, mobilization, and later anabolic needs. Van Zanten et al. (7) similarly argued for the right nutrition, in the right patient, at the right time, rather than a uniform prescription across the illness course.

During post-ICU recovery, nutrition shifts from physiological maintenance to functional rebuilding. Singer (16), Van Zanten et al. (7), and Dupuis and Preiser (17) emphasized that nutrition care should continue beyond ICU discharge because poor intake, weakness, muscle loss, and rehabilitation demands often persist. Thus, the same intake may mean safe initiation during acute instability, deficit correction during prolonged ICU care, or support for muscle preservation and functional recovery during rehabilitation.

### 3.3 Disease phase as the organizing principle of nutritional decision-making

Phase assignment should be repeated after clinically important events. Escalation of vasopressors, a new operation, recurrent sepsis, proning, extubation, transition to intermittent dialysis, or transfer out of the ICU can alter both metabolic demand and route feasibility. A plan appropriate before such an event should not be continued automatically. Reassessment prevents a temporary conservative strategy from becoming chronic underdelivery and prevents a recovery-oriented prescription from being maintained during renewed instability.

**Figure 1:**
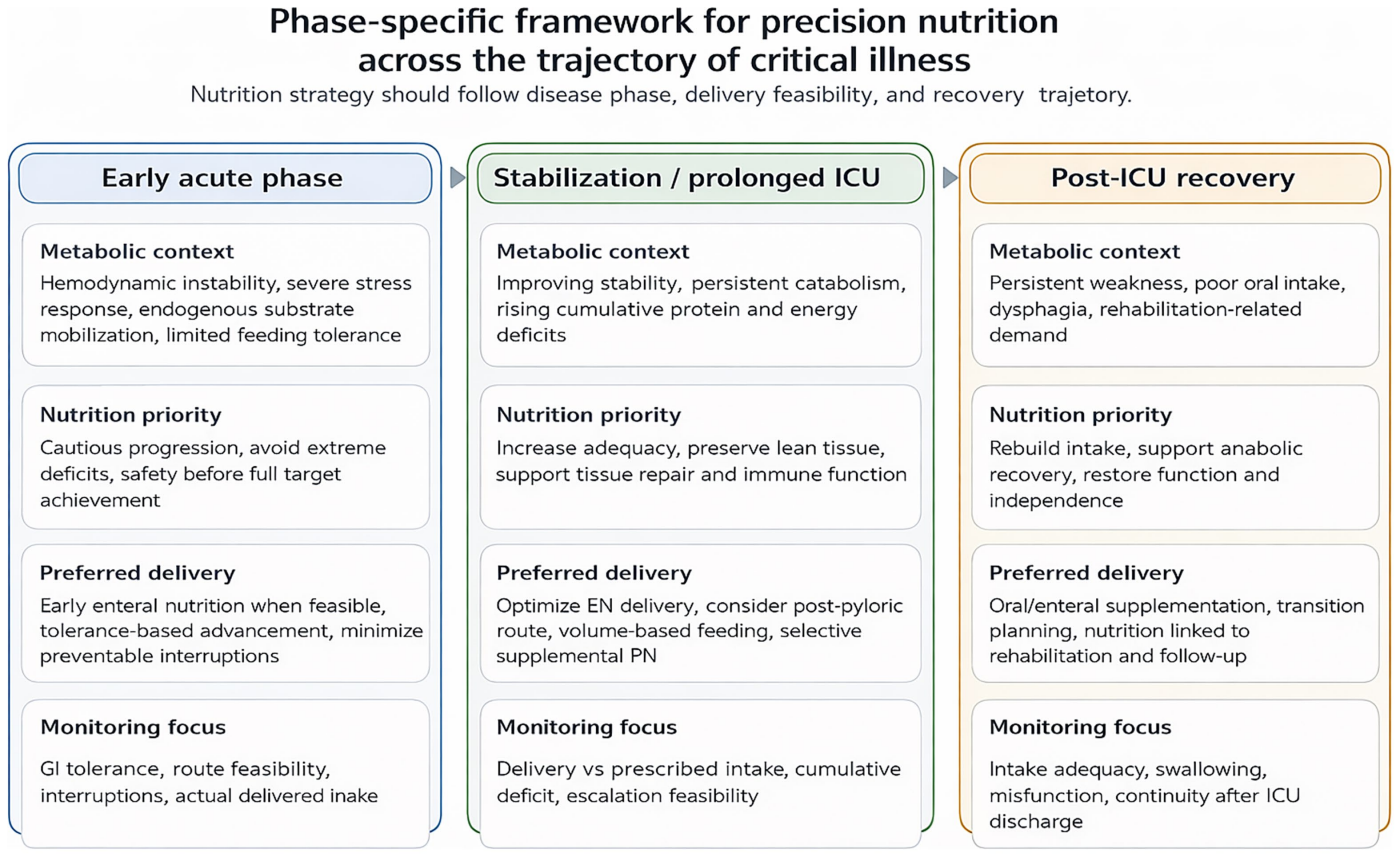
Phase-specific framework for precision nutrition across the trajectory of critical illness. Nutritional priorities evolve from cautious initiation during early acute instability to correction of cumulative deficits during stabilization and to recovery-oriented support after ICU discharge. The framework integrates metabolic context, delivery strategy, and monitoring priorities across phases.

At the bedside, the framework uses four recurring questions: What physiological phase is dominant? How credible are the energy and protein targets? What proportion is actually delivered after interruptions and non-nutritional calories are counted? Is the current route likely to provide adequate exposure within the time available? Figure 1 and Table 1 convert these questions into an operational sequence.

**Table 1:** Phase-specific interpretation of protein and energy targets in critical illness.

Disease phase	Metabolic/clinical context	Protein strategy	Energy strategy	Main clinical goal	Practical caution
Early acute phase	Hemodynamic instability, high stress catabolism, limited gastrointestinal tolerance	Progressive introduction rather than immediate maximal dosing	Cautious advancement rather than forced full replacement	Avoid starvation and extreme deficits while maintaining safety	Do not overinterpret early underdelivery if tolerance and instability dominate
Stabilization/prolonged ICU	Improving stability but persistent catabolism and rising cumulative deficits	Increase adequacy to preserve lean tissue and support repair	Move toward more accurate and consistent delivery; reduce accumulated debt	Protein and energy adequacy become more clinically meaningful	Measure delivered rather than prescribed intake; optimize route feasibility
Post-ICU recovery	Persistent weakness, poor oral intake, dysphagia, rehabilitation demand	Support anabolic recovery and coordinate with mobilization	Rebuild intake and correct unresolved deficits after ICU discharge	Restore oral intake, function, and independence	Ward transition and follow-up failure can perpetuate nutritional debt

## 4 Protein and energy targets across phases of critical illness

### 4.1 Protein targets across phases: from cautious initiation to recovery-oriented support

#### 4.1.1 Biological rationale for protein delivery

Among macronutrients, protein has received particular attention in critical illness because it is closely related to nitrogen balance, lean body mass, wound healing, immune function, and functional recovery. Reignier et al. (6) described critical illness as a dynamic condition shaped by organ failure, inflammation, immobilization, and recovery needs, suggesting that protein requirements should be interpreted according to disease phase rather than as a fixed target. Badpeyma et al. (24) also emphasized that metabolic stress, inactivity, and catabolism promote muscle wasting, making adequate protein biologically plausible for preserving lean tissue.

However, biological plausibility has not translated into a simple rule that higher protein is always better. In a dose–response and pairwise meta-analysis of randomized trials, Badpeyma et al. (24) found that higher protein intake was associated with less muscle atrophy but did not significantly reduce mortality, infections, mechanical ventilation duration, ICU length of stay, or hospital length of stay. Lee et al. (25) likewise noted substantial heterogeneity across trials in protein dose, timing, route, co-delivered energy, patient population, and outcome selection. Heuts et al. (26) further highlighted that average effects of higher versus lower protein delivery may obscure subgroup- or phase-specific responses. Most importantly, the EFFORT Protein trial by Heyland et al. (27) did not show a consistent overall clinical benefit of higher protein dosing in nutritionally high-risk critically ill patients. Therefore, protein delivery should be interpreted as phase-sensitive and phenotype-dependent: cautious during early instability, increasingly relevant during stabilization or prolonged ICU care, and potentially most meaningful when linked to muscle preservation, rehabilitation participation, and functional recovery.

#### 4.1.2 Why trial results remain inconsistent

Trial results on protein delivery remain inconsistent partly because early critical illness may not be the phase in which higher exogenous protein is most efficiently used. Reignier et al. (6) described early critical illness as a state dominated by acute organ failure, inflammation, immobilization, and changing recovery needs, while Patel et al. (12) emphasized that early nutrition should remain feasible and tolerance-based rather than purely target-driven. This provides a physiological explanation for why the EFFORT Protein trial by Heyland et al. (27) did not show a consistent overall benefit from higher protein dosing, even in nutritionally high-risk patients. Haines et al. (28), in a secondary Bayesian analysis of EFFORT Protein, further suggested that average trial effects may conceal heterogeneity across patient subgroups and illness phases rather than proving that protein dose is irrelevant. Another reason is that the prescribed protein dose may differ substantially from the delivered dose. Heuts et al. (26) emphasized uncertainty around higher versus lower protein delivery and highlighted that treatment effects may vary according to timing, patient phenotype, and achieved exposure. Tweel et al. (29) specifically examined early and late acute-phase protein dose and time-to-discharge-alive, reinforcing that protein exposure should be interpreted dynamically rather than as a single average ICU value. Accordingly, the distinction between “high” and “lower” protein groups in clinical trials may be smaller in real exposure than it appears on paper (25, 27, 29).

Finally, conventional ICU outcomes may not match the hypothesized mechanism of protein therapy. Lee et al. (25) found that higher protein delivery did not improve mortality or other major clinical outcomes, although it attenuated muscle loss in smaller studies. Badpeyma et al. (24) similarly reported no clear reduction in mortality, infection, mechanical ventilation duration, ICU stay, or hospital stay, despite biologically plausible links between protein and lean mass preservation. Qin et al. (30) and Blaauw et al. (31) also questioned whether higher or guideline-recommended protein delivery consistently improves mortality or length-of-stay outcomes. Thus, inconsistent findings may reflect a mismatch between mechanism, timing, delivered exposure, and selected endpoints rather than a simple conclusion that protein is unimportant.

#### 4.1.3 A phase-specific interpretation of protein targets

The absence of a uniform benefit from higher protein delivery should not be interpreted as evidence that protein is unimportant. Van Zanten et al. (7) emphasized that nutrition therapy should extend across the ICU, post-ICU, and long-term recovery phases, with different priorities as patients move from survival to rehabilitation and functional rebuilding. Patel et al. (12) similarly supported the early use of enteral nutrition when feasible, but their argument is better understood as support for safe and progressive delivery rather than immediate high-dose protein replacement in all patients. Therefore, the more relevant question is not whether higher protein is always superior, but when protein becomes clinically meaningful and in which patient phenotype (7, 12, 23).

Patient phenotype is therefore central to any interpretation of protein targets. A young patient with short ICU exposure and preserved nutritional reserve is unlikely to have the same vulnerability to protein deficit as an older patient with frailty, pre-existing malnutrition, obesity with low muscle reserve, or prolonged multiorgan failure. Wischmeyer et al. (1) emphasized that protein provision should be adjusted according to clinical context and tolerance rather than applied as a uniform target. Patel et al. (12) similarly framed nutrition delivery as a practical and individualized process, particularly when gastrointestinal function, hemodynamic stability, and delivery feasibility are uncertain. Burns, trauma, large wounds, and renal replacement therapy may further modify protein losses, metabolic demand, or delivery priorities, making these populations biologically distinct from short-stay, lower-risk ICU patients (32). Yet many randomized trials and evidence syntheses aggregate these phenotypes, which may dilute subgroup-specific effects and contribute to neutral average results (24, 25, 33, 34).

A second issue is that prescribed protein is not the same as delivered protein. Heuts et al. (26) highlighted uncertainty around higher versus lower protein delivery and suggested that treatment effects may depend on timing, patient phenotype, and achieved exposure. The EFFORT Protein trial by Heyland et al. (27) tested higher protein dosing in nutritionally high-risk patients but did not show a consistent overall benefit, raising the possibility that patient selection, timing, and actual exposure may be more important than the nominal target alone. Tweel et al. (29) further examined early and late acute-phase protein exposure, supporting the view that protein should be analyzed dynamically rather than as one average ICU dose. Future trials should therefore document real protein delivery, interruptions, co-delivered energy, and phase-specific exposure rather than relying only on prescribed targets (26, 27, 29).

The timing of protein advancement also requires caution. Reignier et al. (6) described early critical illness as a dynamic state shaped by organ failure, inflammation, immobilization, and evolving recovery needs, while Patel et al. (12) emphasized feasibility and tolerance in early feeding. These considerations help explain why immediate escalation to high protein doses may not be appropriate during unstable acute illness, especially before hemodynamic stability, gastrointestinal tolerance, and adequate energy delivery are established (6, 12, 27, 28). A more balanced interpretation is staged progression: cautious initiation during early instability, active reassessment as shock and gastrointestinal dysfunction resolve, and more assertive protein provision when the patient enters stabilization, prolonged ICU care, or recovery (1, 6, 7, 12).

Protein targets should also be linked to implementation and rehabilitation. A high-protein prescription may have little clinical meaning if enteral nutrition is frequently interrupted, protocol adherence is poor, or supplemental strategies are not used when intake remains inadequate (1, 9, 26, 27, 29). The clinical question is therefore not only “what dose should be prescribed?” but also “how can reliable delivery of the prescribed dose be ensured?” Precision nutrition requires that protein targets be paired with implementation strategies, otherwise the difference between intention and exposure may remain too large to matter (35–37). Similarly, protein delivery alone may not restore muscle if the patient remains profoundly inactive, whereas rehabilitation may be less effective without adequate amino acid availability (7, 16, 17, 38). Thus, precision nutrition should pair protein targets with reliable delivery systems and recovery-oriented rehabilitation pathways. Overall, protein should be interpreted as phase-sensitive, phenotype-dependent, and delivery-dependent rather than as a fixed universal target (6, 7, 12, 23).

### 4.2 Energy targets across phases: assessment, timing, and cumulative deficit

#### 4.2.1 Predictive equations versus indirect calorimetry

Energy management in critically ill patients has long been dominated by the question of target achievement: how many calories should be delivered, and how rapidly should that goal be reached? Yet this framing obscures two separate problems. The first is how energy needs are estimated; the second is whether the patient’s current physiological phase warrants immediate full replacement of the calculated target. Confusion between these two issues has contributed to persistent controversy (39–43). Singer et al. (3, 4) emphasized in the ESPEN guideline that measured energy expenditure is preferable when indirect calorimetry is available, because predictive equations are often inaccurate in critically ill patients. However, this recommendation supports more accurate assessment; it does not imply that every measured target should be fully achieved immediately during early acute illness.

Predictive equations remain widely used because they are simple and available, but their accuracy is limited by the dynamic physiology of critical illness. Fever, sepsis, sedation, mechanical ventilation, extracorporeal support, obesity, fluid accumulation, and changes in activity can all alter resting energy expenditure (41, 42). Zusman et al. (44) and related studies showed that discrepancies between estimated and measured expenditure may be clinically important, especially during prolonged ICU stay or metabolic instability (41–43, 45). Thus, equations may be useful as rough estimates when indirect calorimetry is unavailable, but they are a weak basis for strong conclusions about underfeeding or overfeeding.

Indirect calorimetry improves this problem by measuring energy expenditure from oxygen consumption and carbon dioxide production rather than relying on formula-based assumptions (39–43). Meta-analyses suggest that indirect calorimetry-guided feeding can improve the precision of energy prescription, but its effects on mortality, infection, length of stay, or ventilator outcomes remain inconsistent (39, 40). The TICACOS trial and subsequent studies illustrate this distinction. TICACOS tested indirect calorimetry-guided nutritional support and suggested that measured expenditure can be used to individualize caloric delivery, but the trial was limited by sample size and was not definitive for hard clinical endpoints (43). Later trials and discussions of energy-target studies, including evaluations of TARGET and later energy-target studies, further showed that simply pursuing higher or more accurate calorie targets does not necessarily improve outcomes if timing, disease phase, protein delivery, and patient phenotype are not considered (44, 46).

Therefore, indirect calorimetry should be interpreted as a tool for improving physiological assessment, not as a stand-alone intervention that is universally beneficial in all ICU patients. In early acute instability, even a measured target may require cautious advancement. During stabilization or prolonged ICU care, measured expenditure may help prevent cumulative energy debt. During recovery, repeated assessment may help align energy delivery with increasing activity, rehabilitation, and anabolic needs (39–43). This distinction supports a shift from protocol-driven feeding toward physiology-informed, phase-sensitive energy delivery.

#### 4.2.2 Early energy delivery and metabolic tolerance

Once the credibility of the energy target is considered, the next question is timing. Randomized trials have challenged the assumption that calculated energy deficits should be corrected as early as possible. In the PermiT trial, Arabi et al. (21) showed that permissive underfeeding did not worsen survival compared with standard feeding in critically ill adults. Rice et al. (22) similarly found that initial trophic feeding was not clearly inferior to full enteral feeding for major short-term outcomes in mechanically ventilated patients with acute lung injury or acute respiratory failure. These trials do not prove that underfeeding is beneficial, but they suggest that immediate full caloric replacement during early acute illness is not consistently superior to a more conservative strategy (21, 22). The goal in early illness may be better described as controlled progression rather than immediate total replacement of all theoretical deficits (6, 12, 42).

However, early restraint should not become prolonged underfeeding. The permissive and trophic feeding trials mainly inform the early acute phase and short-term ICU outcomes (21, 22). Van Zanten et al. (7) and Singer (16) emphasized that after ICU survival, nutritional priorities shift toward preventing persistent deficits, supporting rehabilitation, and improving post-ICU recovery (17). Thus, the significance of underfeeding depends on phase and duration: it may be acceptable during acute instability but harmful if it persists during stabilization or recovery (7, 16, 17).

Energy provision also depends on feasibility. A prescribed calorie goal has limited meaning if feeding interruptions, gastric intolerance, procedures, fluid restriction, prone positioning, or vasopressor-dependent instability prevent actual delivery. Energy-target trials and discussions, including TICACOS (43), TARGET (46), later energy-target studies and related analyses, suggest that more precise or higher calorie targets do not automatically improve outcomes unless timing, tolerance, phenotype, and delivery feasibility are considered (39, 44). Precision energy delivery should therefore link target definition, route feasibility, tolerance monitoring, actual intake, and disease phase rather than focus only on the numerical calorie prescription (42).

#### 4.2.3 When cumulative deficits become clinically relevant

Energy targets should not be judged only by short-term ICU outcomes. The PermiT and trophic-feeding trials showed that conservative early energy delivery was not clearly inferior to standard or full feeding for major short-term outcomes (21, 22). The TARGET trial also failed to show a survival benefit from higher energy delivery using energy-dense enteral nutrition (46). These findings challenge routine early full caloric replacement, but they do not prove that persistent energy deficits are harmless throughout prolonged ICU care or post-ICU recovery (21, 22, 46). The real issue is how to avoid both unnecessary early overfeeding and passive continuation of inadequate intake after stabilization (6, 17, 20).

The clinical meaning of energy deficit depends on phase, duration, and patient vulnerability. Reignier et al. (6) described nutritional priorities as shifting from acute organ failure and early tolerance constraints toward recovery, progressive intake, and mobilization. Van Zanten et al. (7) and Singer (16) further emphasized that inadequate intake, muscle loss, and rehabilitation needs often persist after ICU survival (17). Thus, a short deficit during severe shock is not equivalent to a deficit that continues through prolonged ventilation, inflammation, tissue injury, or recovery (6, 7, 12, 16, 17). Energy management also depends on whether the target can actually be delivered. Volume-based feeding and protocolized approaches may reduce avoidable underdelivery, but their effect depends on workflow, interruptions, tolerance assessment, and staff adherence (36, 37). An ambitious calorie target may therefore create only the appearance of adequate nutrition if bedside delivery remains inconsistent (36, 37, 42).

## 5 Feeding intolerance as a barrier to meaningful nutrient delivery

### 5.1 Definitions, heterogeneity, and clinical consequences

Feeding intolerance is central to critical care nutrition because it determines whether planned enteral nutrition can actually be delivered. Li et al. (13) showed that different definitions of feeding intolerance lead to different interpretations of prevalence and outcomes in critically ill adults receiving enteral nutrition. Jenkins et al. (14) similarly reported substantial variation in definitions, including gastric residual volume, vomiting, diarrhea, abdominal distension, bowel sounds, aspiration events, enteral nutrition interruption, or failure to reach nutritional targets. Reintam Blaser et al. (15) further showed that feeding intolerance is associated with adverse outcomes, but comparison across studies is limited by definitional heterogeneity.

This heterogeneity affects bedside decisions. A narrow definition based on one marker, such as gastric residual volume, may lead to unnecessary interruption of enteral nutrition, whereas overly restrictive criteria may miss true gastrointestinal dysfunction or persistent delivery failure (13–15). Therefore, this review uses a functional definition: feeding intolerance refers to gastrointestinal dysfunction or feeding-related symptoms that materially prevent sustained enteral nutrition delivery and achievement of intended nutritional goals. This is a pragmatic, delivery-oriented framework rather than a universally validated consensus definition (13–15).

### 5.2 Risk factors and prediction challenges

Feeding intolerance is more frequent in patients with greater physiological stress. Risk-factor studies have associated it with high illness severity, shock, vasopressor exposure, deep sedation, opioid use, mechanical ventilation, prone positioning, abdominal pathology, and pre-existing gastrointestinal dysfunction (47, 48). These studies support the view that feeding intolerance often reflects systemic critical illness rather than isolated digestive dysfunction, but most evidence is observational and cannot prove that any single factor independently causes intolerance (47, 48).

Prediction models may help identify high-risk patients, but systematic reviews show important limitations, including inconsistent outcome definitions, variable predictor selection, incomplete external validation, and uncertain generalizability across ICU populations (49–51). Therefore, these tools should be viewed as aids to risk awareness rather than definitive decision-making instruments.

Low enteral nutrition delivery is also not always caused by true gastrointestinal dysfunction. Procedures, transport, prone positioning, airway manipulation, hemodynamic instability, and aspiration concerns can interrupt feeding even when gastrointestinal symptoms are limited (47, 48). This distinction matters because avoidable interruptions require workflow solutions, whereas true gastrointestinal dysfunction may require promotility therapy, post-pyloric feeding, formula adjustment, or supplemental strategies.

Overall, feeding intolerance should be interpreted as a multidimensional delivery-limiting condition that includes gastrointestinal dysfunction, avoidable feeding interruptions, aspiration risk, and persistent inability to sustain enteral nutrition. This framework is clinically useful but remains pragmatic rather than a validated diagnostic algorithm (13–15, 48–51). Table 2 translates this multidimensional interpretation into clinically usable categories by linking gastrointestinal dysfunction, avoidable interruptions, aspiration risk, and failure of sustained EN delivery with corresponding assessment and management implications.

**Table 2:** Feeding intolerance in critical illness: definitions, consequences, and management implications.

Domain	Key points	Clinical implication
Definitional heterogeneity	Studies variably use gastric residuals, vomiting, diarrhea, abdominal distension, aspiration events, or inability to achieve intended EN delivery.	A broader delivery-oriented definition is more clinically useful than any single sign.
Common manifestations	Vomiting/regurgitation, retention, distension, diarrhea, repeated feeding interruption, and poor progression of EN.	Symptoms should be interpreted together with whether planned delivery can actually be sustained.
Major risk factors	Shock, vasopressors, deep sedation, opioids, mechanical ventilation, prone positioning, abdominal pathology, and high illness severity.	The sickest patients are often both the most nutritionally vulnerable and the most difficult to feed.
Why intolerance matters	Actual exposure often diverges from prescription because symptoms and ICU workflow reduce delivery reliability.	Failure of delivery should trigger reassessment of route, protocol, and escalation strategy.
Strategies to preserve EN	Reduce avoidable interruptions, consider continuous infusion or volume-based feeding, optimize aspiration precautions, and use promotility measures when appropriate.	The first goal is usually to preserve EN by improving the way it is delivered.
When to escalate route or support	Persistent gastric failure may justify post-pyloric feeding; unresolved clinically meaningful deficits may justify selective supplemental PN.	Escalation decisions should be phase-sensitive and based on expected duration of EN inadequacy.

### 5.3 Optimizing enteral nutrition under feeding intolerance

#### 5.3.1 Gastric versus post-pyloric feeding

When enteral nutrition is poorly tolerated, the priority is usually to preserve EN by improving delivery rather than abandoning it. Patel et al. (12) and Reignier et al. (6) support EN as the preferred route when feasible, but not as an inflexible approach when delivery repeatedly fails. Gastric feeding is simple and appropriate as the initial route. However, persistent vomiting, high gastric retention, recurrent interruptions, or aspiration concern may justify post-pyloric feeding (35, 52). Evidence suggests post-pyloric access may improve delivery in selected patients, but benefits vary with patient selection, placement success, local expertise, and definitions of intolerance (35, 53). Thus, post-pyloric feeding should not be routine for all ICU patients, but should be considered when gastric feeding failure becomes persistent and delivery-limiting (35, 36, 52, 53).

#### 5.3.2 Continuous versus intermittent feeding

Continuous and intermittent feeding should be selected according to tolerance, workflow, and disease phase. Panwar et al. (54) found that trials comparing these strategies were heterogeneous in populations, protocols, intolerance definitions, and outcomes, so neither approach is universally superior. Continuous feeding may be more suitable during early instability or high intolerance risk, whereas intermittent or cyclic feeding may be considered later as gastrointestinal function, mobility, and oral intake improve (54).

#### 5.3.3 Volume-based feeding and protocol optimization

Enteral nutrition often underdelivers because of repeated interruptions rather than route choice alone. Wang et al. (36) showed that volume-based feeding can improve EN adequacy by shifting from a fixed hourly rate to a 24-h volume target, but its success depends on safety limits, tolerance monitoring, nursing workload, and protocol adherence (37). Thus, VBF is an implementation tool to reduce avoidable underdelivery, not evidence that higher targets alone improve outcomes. Supportive measures such as intolerance assessment, promotility therapy, aspiration-risk reduction, post-pyloric feeding, and multidisciplinary review should be matched to the cause of underdelivery (52, 55, 56).

In summary, optimizing EN under intolerance requires more than choosing a formula or setting a rate. It involves deliberate adaptation of route, infusion pattern, and bedside processes to the patient’s tolerance profile and the operational realities of the ICU. Gastric feeding, post-pyloric feeding, continuous infusion, intermittent strategies, and VBF should all be viewed as tools within a broader enteral delivery strategy. The best approach is the one that sustains safe and meaningful nutrient delivery in the patient’s current clinical context (35, 36, 52).

### 5.4 Supplemental parenteral nutrition as a compensatory strategy

#### 5.4.1 Early versus late supplemental PN

When EN remains insufficient, the key question is not simply early versus late PN, but whether clinically meaningful deficits cannot be corrected enterally. CALORIES showed broadly similar major outcomes with early PN and early EN, suggesting that PN can be feasible when EN is not possible (57). In contrast, EPaNIC found worse short-term recovery signals with early supplemental PN, warning against premature PN escalation during early acute illness (58). Meta-analyses suggest that supplemental PN can improve energy and protein delivery when EN is inadequate, but effects on mortality, infection, and length of stay remain inconsistent (58–61). Thus, better nutrient delivery does not automatically mean better outcomes, and risks such as overfeeding, hyperglycemia, infection, and fluid burden must be considered (58–62).

A phase-sensitive interpretation is therefore preferable: tolerate limited early underdelivery during acute instability, but consider supplemental PN during stabilization or prolonged ICU care when EN failure persists, deficits accumulate, and nutritional risk is high (58–62).

#### 5.4.2 Permissive underfeeding and phase-based interpretation

Permissive underfeeding should be interpreted as a conditional early-phase strategy, not as a general preference for low intake. Arabi et al. (21) and Rice et al. (22) showed that conservative or trophic feeding was not clearly inferior to standard or full feeding for major short-term ICU outcomes. However, these trials mainly support limited early restraint during acute instability; they do not justify prolonged underdelivery after stabilization (21, 22).

Supplemental PN should therefore be viewed as one tool within a staged feeding strategy rather than as an alternative ideology to EN. EPaNIC cautions against premature early supplemental PN, whereas later evidence and reviews suggest that PN may help correct clinically meaningful deficits when EN remains persistently inadequate. Thus, PN may be unnecessary if EN is improving and adequacy is likely soon, but continued avoidance of PN may be inappropriate in patients with repeated EN failure, high nutritional risk, and prolonged ICU exposure (58, 59, 63).

#### 5.4.3 When unresolved deficits justify escalation

Starting PN does not mean fully replacing every estimated deficit. Early trials such as PermiT caution against immediate complete caloric correction during acute instability (21). Meta-analyses suggest that supplemental PN can improve energy and protein delivery, but clinical outcome benefits remain inconsistent (59, 62). EPaNIC and subsequent analyses also caution against premature early PN escalation (58, 60). Therefore, PN should be titrated by phase, tolerance, remaining EN delivery, and nutritional risk.

**Figure 2:**
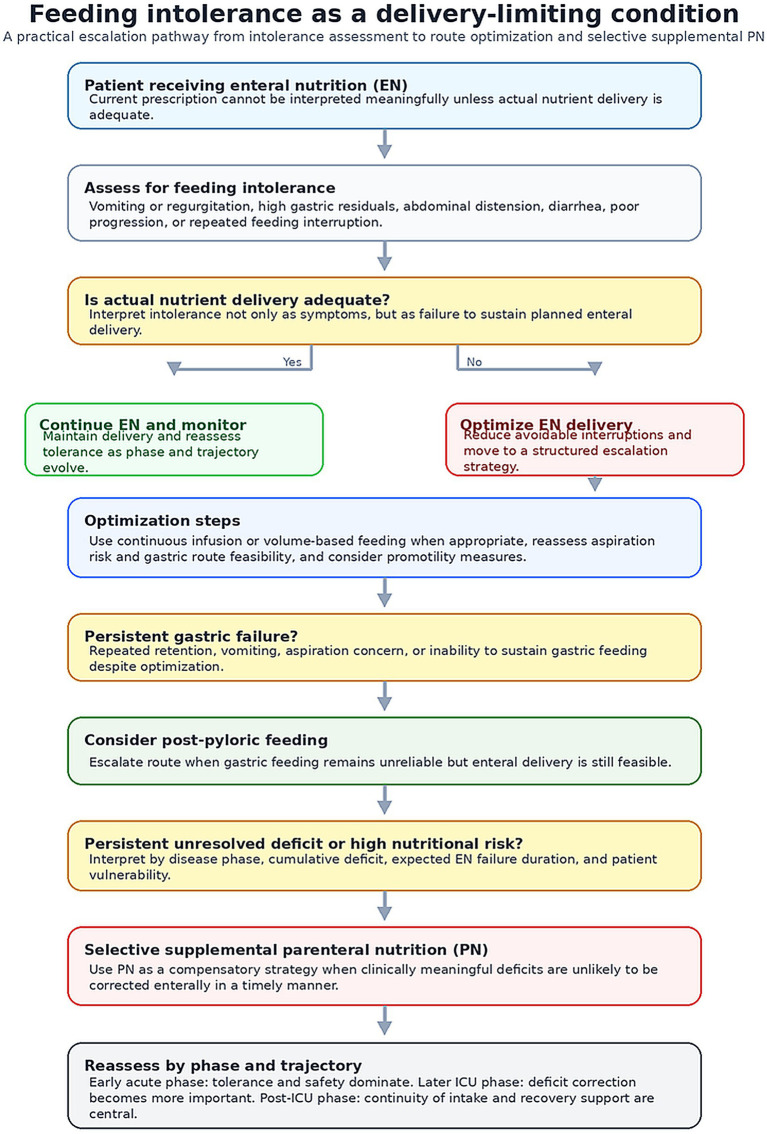
Feeding intolerance as a delivery-limiting condition: a practical escalation pathway. Feeding intolerance should be interpreted according to its effect on sustained enteral nutrition delivery rather than by symptoms alone. The pathway summarizes a stepwise approach from assessment of intolerance and delivery failure to optimization of enteral feeding, route escalation, and selective use of supplemental parenteral nutrition.

PN-related risks, including infection, hyperglycemia, overfeeding, and fluid burden, remain important (58, 60, 64). However, these risks support selective use, not complete avoidance. Supplemental PN is best viewed as a phase-sensitive bridging strategy when EN failure persists and clinically meaningful deficits accumulate in high-risk patients (21, 57, 58, 62). Figure 2 and Table 2 summarize this delivery-centered approach.

## 6 Nutrition beyond ICU survival: recovery, transition, and functional rebuilding

### 6.1 Ongoing nutritional vulnerability after ICU discharge

Post-ICU nutrition has become increasingly important because survival from critical illness does not necessarily mean resolution of nutritional risk. Singer (16) described the post-ICU trajectory as a distinct phase in which nutritional care should continue beyond acute ICU support, since patients may remain vulnerable despite clinical stabilization. Van Zanten et al. (7) similarly argued that nutrition therapy should extend from ICU care to post-ICU and long-term convalescence, with recovery-oriented goals replacing purely survival-oriented targets. These reviews support the concept of ongoing nutritional vulnerability, but they are mainly expert and narrative syntheses rather than definitive trials of specific post-ICU nutrition protocols (7, 16).

Post-ICU vulnerability reflects a mismatch between ongoing needs and reduced delivery. Dupuis and Preiser (17) emphasized that recovery requires a personalized, multimodal approach because muscle loss, weakness, inflammation, poor appetite, and rehabilitation demands often coexist. Terblanche et al. (18) highlighted barriers such as dysphagia, fatigue, anorexia, psychological symptoms, and discontinuity of care. Rosseel et al. (19) showed that ICU survivors on general wards frequently fail to achieve adequate energy and protein intake. Thus, the ward phase is not nutritionally neutral, although evidence remains heterogeneous in assessment methods, targets, and interventions (17–19).

Post-ICU deficits may also be generated after ICU discharge when nutrition support is reduced, oral intake is overestimated, follow-up becomes less intensive, or rehabilitation increases demand (16–19). Nienow et al. (65) emphasized the need to prioritize nutrition during recovery, but no universal target, follow-up interval, or intervention package is yet validated for all ICU survivors. Therefore, post-ICU nutrition should be viewed as a high-risk transition requiring structured reassessment rather than an automatic return to routine oral intake.

### 6.2 Functional consequences of persistent deficits

Persistent post-ICU nutritional deficits may contribute to muscle wasting, ICU-acquired weakness, reduced mobility, delayed respiratory recovery, and poor rehabilitation tolerance. Dupuis and Preiser (17) emphasized that recovery after critical illness requires a multimodal approach linking nutrition, muscle, function, and rehabilitation. Terblanche et al. (18) also highlighted that poor intake after critical illness may worsen weakness and recovery barriers. However, these data mainly support clinical relevance rather than proving that one specific nutrition strategy improves all functional outcomes. This imbalance may have contributed to the underappreciation of nutrition’s role in later recovery (66).

Body weight alone is also insufficient to assess recovery. Singer (16) emphasized that post-ICU nutrition should be evaluated beyond survival and basic intake, while muscle-focused literature shows that fluid shifts and body composition changes may mask persistent lean mass loss (67). Thus, recovery-oriented nutrition should be linked to strength, activity, rehabilitation participation, and functional outcomes, not only calories or weight.

Post-ICU recovery may be a therapeutic window because nutrition may better support anabolism once acute instability begins to resolve, especially when combined with rehabilitation (16, 17, 65). This remains a plausible but incompletely validated pathway, so future studies should test nutrition-rehabilitation strategies using patient-centered outcomes such as muscle preservation, independence, quality of life, and daily function (17, 66).

### 6.3 Recovery-phase nutrition as organized rehabilitation

Recovery-phase nutrition should be organized as part of rehabilitation rather than treated as passive return to oral intake. Dupuis and Preiser (17) emphasized that recovery after critical illness requires a personalized, multimodal approach, while Nienow et al. (65) argued that nutrition should be prioritized during recovery rather than assumed to normalize spontaneously. However, these papers support a care model rather than a validated universal protocol.

Continuity across transitions is central. Post-ICU and ward studies show that intake often remains inadequate after ICU discharge, while dietetic support may weaken during transfer (19, 20, 68). Therefore, dysphagia assessment, texture modification, oral nutritional supplements, assisted feeding, symptom control, patient education, and multidisciplinary coordination are essential to make nutrition plans survive ICU-to-ward handover (20, 69, 70).

During recovery, nutrition shifts from preventing severe deficit to supporting functional rebuilding. Singer (16), Van Zanten et al. (7), and Dupuis and Preiser (17) linked post-ICU nutrition with muscle preservation, rehabilitation participation, and functional recovery. This rationale is clinically important but still incompletely validated, so future studies should test integrated nutrition-rehabilitation pathways using strength, independence, quality of life, and long-term recovery as outcomes (16, 17, 65).

### 6.4 Nutrition across care transitions, family support, and follow-up systems

The ICU-to-ward transition can create new nutritional risk rather than simply reveal residual deficits from critical illness. Rosseel et al. (19) showed that ICU survivors on general wards often fail to meet energy and protein targets, while studies of post-ICU nutrition and recovery pathways highlight reduced monitoring, weaker dietetic continuity, and overestimation of oral intake after ICU discharge (20, 68). These findings suggest that post-ICU nutritional decline is partly preventable through better transitional care, although the evidence mainly supports service improvement rather than one validated follow-up model.

A critical issue is that clinical stability does not equal nutritional recovery. Terblanche et al. (18) emphasized that ICU survivors may continue to experience poor appetite, dysphagia, fatigue, psychological symptoms, and functional barriers to eating. Long-term outcome literature also shows that recovery should be assessed through strength, mobility, independence, and quality of life, not only survival or discharge readiness (66). Therefore, nutrition should be considered part of survivorship care rather than a narrow ICU feeding metric.

Family and social context also influence whether nutrition plans are implemented after discharge. ICU survivors may struggle with shopping, cooking, meal pacing, texture modification, oral supplements, and symptom monitoring, and informal caregivers may require guidance to support adequate intake (18, 71). However, this area remains underdeveloped in interventional research, so family-centered nutrition support should be viewed as a practical priority requiring further evaluation rather than an established standardized intervention.

Follow-up systems may help identify persistent nutritional risk after discharge. Post-ICU clinics and structured recovery programs provide opportunities to reassess intake, weight change, body composition, symptoms, and rehabilitation progress (20, 66). Yet such systems are not consistently available, and the optimal timing, content, and intensity of nutrition follow-up remain uncertain. Recovery-phase nutrition therefore needs its own operational indicators, including restoration of oral intake, maintenance or recovery of muscle, rehabilitation participation, and reduction of nutrition-related barriers to discharge (17, 69). Table 3 summarizes major post-ICU nutritional problems, suggested assessments, and practical response strategies for recovery-oriented care.

**Table 3:** Nutritional priorities after ICU discharge: problems, assessment tools, and care strategies.

Post-ICU problem	Why it matters	Suggested assessment	Nutritional/clinical response
Poor oral intake	Calorie and protein intake often remain far below needs despite apparent clinical improvement.	Dietary intake review, supplement use, bedside nutrition assessment.	Structured oral supplementation, dietitian review, continuation of enteral support when needed.
Dysphagia	Swallowing impairment limits safe oral intake and may delay recovery.	Swallowing assessment and texture review.	Texture adaptation, speech-language support, temporary tube feeding when required.
Persistent cumulative deficit	ICU underdelivery may continue or worsen after transfer to the ward.	Trajectory review of delivered intake and unresolved deficits.	Active correction plan rather than passive assumption of spontaneous recovery.
Skeletal muscle loss / weakness	Functional recovery may lag behind apparent medical stabilization.	Muscle ultrasound or body composition tools when available; functional assessment.	Link protein/energy support with mobilization and rehabilitation.
Inadequate care transition	Nutrition plans often weaken during handover from ICU to ward or home.	Review of handover documentation and tube/oral feeding plan.	Explicit transition plan with responsibilities, targets, and follow-up.
Lack of follow-up	Late nutritional deterioration may go unnoticed until weight loss or readmission occurs.	Post-ICU clinic review, intake symptom review, functional reassessment.	Embed nutrition into survivorship and recovery pathways.

## 7 Monitoring recovery: metabolic demand, muscle trajectory, and implementation tools

### 7.1 Indirect calorimetry in the recovery phase

Recovery-phase precision nutrition requires tools that detect persistent metabolic risk beyond clinical impression. Singer (16) emphasized that nutritional vulnerability may continue after ICU discharge, while Kamel et al. (42) noted that energy expenditure is difficult to estimate and may change across illness phases. Thus, indirect calorimetry may help identify patients whose requirements remain elevated during recovery. However, evidence that routine recovery-phase indirect calorimetry improves outcomes remains limited. Feasibility, cost, availability, and timing of repeated measurements are also unresolved. Therefore, indirect calorimetry should be viewed as a promising assessment tool for selected high-risk recovery patients, not as a mandatory standard for all ICU survivors.

### 7.2 Muscle monitoring beyond body weight

Muscle monitoring is important because body weight and body mass index are poor markers of recovery in critical illness, especially when fluid shifts and edema obscure lean tissue loss. Muscle-focused reviews suggest that bedside ultrasound is feasible, repeatable, and potentially useful for tracking muscle trajectory in ICU patients, but its use remains limited by variation in technique, measurement site, operator training, and clinically validated thresholds (67, 72).

Therefore, muscle ultrasound should be viewed as a promising monitoring tool rather than a fully standardized outcome measure. Its value lies in showing whether nominal nutrition delivery is accompanied by preservation or recovery of functionally relevant tissue. If muscle continues to decline despite apparent intake adequacy, clinicians should reassess protein delivery, rehabilitation participation, inflammation, and actual nutrient exposure (16, 67, 72).

### 7.3 Digital tools and operational precision

Digital and organizational tools may support longitudinal precision nutrition, but their current role remains adjunctive. Post-ICU follow-up systems can help reassess nutrition, symptoms, function, and rehabilitation progress after discharge (20). Artificial intelligence and decision-support tools may integrate intake, metabolic data, feeding tolerance, muscle trajectory, and clinical risk, but current evidence mainly supports their potential for monitoring and coordination rather than proven outcome improvement (73).

Their value depends on data quality and care integration. Dashboards or algorithms cannot compensate for inaccurate intake records, weak follow-up, or lack of clinical response. Digital monitoring may help keep nutrition visible during recovery, especially when intake remains low after ICU discharge (68). Thus, these tools should be viewed as implementation supports, not validated stand-alone interventions (20, 68, 73).

## 8 Research gaps and priorities for phase-specific precision nutrition

### 8.1 Heterogeneity and the problem of average effects

The lack of a universal nutrition strategy does not mean nutrition is irrelevant; it reflects ICU population heterogeneity and trial-design limitations. Patients differ in frailty, baseline function, nutritional risk, obesity, body composition, organ dysfunction, inflammation, and recovery trajectory (66, 69). When analyzed as one group, average treatment effects may hide important subgroup or phase-specific responses. Future studies should therefore move beyond broad ICU categories and test phase-defined, phenotype-aware nutrition strategies using recovery-relevant outcomes, not only mortality or length of stay.

### 8.2 Differential vulnerability in special populations

Special populations show why average ICU trial effects may be misleading. Frailty, low muscle reserve, obesity with sarcopenia, renal replacement therapy, burns, trauma, sepsis, liver dysfunction, and chronic respiratory failure may alter protein losses, energy needs, and delivery feasibility (7, 17, 32). Because many trials are not powered for subgroup effects, a strategy that appears neutral overall may still matter in selected phenotypes. Future studies should stratify patients by nutritional reserve, body composition, organ support, diagnosis, and recovery phase (7, 17, 32).

### 8.3 Limits of current phase definitions, exposure measurement, and endpoints

Current evidence is limited by inconsistent phase definitions. Reignier et al. (6) and Patel et al. (12) both emphasize that nutritional decisions should reflect changing physiology, tolerance, and recovery needs, but terms such as “early,” “late,” “stable,” and “recovery” are often used without standardized clinical criteria. As a result, studies may appear to test the same phase while enrolling patients at different physiological points, complicating interpretation of protein, energy, and PN timing evidence.

Exposure measurement is another weakness. Many studies report prescribed calories or protein more clearly than actual delivered intake, despite the fact that biological exposure depends on what patients receive. Rosseel et al. (19) showed that post-ICU patients often fail to meet energy and protein targets, while implementation studies of volume-based feeding show how interruptions and workflow can dilute intended delivery (36). Future trials should therefore document delivered intake, interruptions, route changes, supplemental strategies, and phase-specific exposure rather than comparing prescriptions alone (19, 36, 69).

Finally, endpoints remain too short-term. Mortality, infection, ventilator days, and ICU length of stay are important, but they may miss benefits most plausibly linked to nutrition, especially during stabilization and recovery. TARGET and long-term outcome literature show the need to include muscle mass, strength, mobility, independence, readmission, quality of life, and other patient-centered recovery outcomes in future nutrition trials (46, 66, 69).

### 8.4 Priorities for future research

Future research should move from broad average effects toward testable, phase-specific questions. First, trials should phenotype patients more carefully by nutritional risk, frailty, body composition, organ support, diagnosis, and illness phase (17, 69). Second, studies should distinguish prescribed from delivered nutrition and report interruptions, route changes, supplemental strategies, and phase-specific exposure, because protocol intention may differ substantially from biological intake (20, 37, 68). Third, outcomes should extend beyond mortality, infection, and length of stay to include muscle mass, strength, mobility, independence, quality of life, readmission, and return to daily function (16, 17, 69).

Future work should also study nutrition as part of recovery care rather than as an isolated ICU intervention. Combined nutrition-rehabilitation pathways, swallowing management, family support, and post-discharge follow-up need prospective evaluation, particularly across ICU-to-ward and hospital-to-home transitions (17, 20, 69). Indirect calorimetry, muscle ultrasound, and digital monitoring platforms should be tested as components of decision pathways, not merely as measurement tools (38, 73).

Implementation research is equally important. Many failures in ICU nutrition reflect delivery architecture rather than disagreement about targets: preventable interruptions, weak handover, poor intake documentation, and inconsistent protocol adherence (20, 37, 68). Future trials and quality-improvement studies should therefore ask which patients, in which phase, under which delivery conditions, benefit from which nutrition strategy. This would make phase-specific precision nutrition a testable and clinically actionable approach rather than only a conceptual framework (17, 69).

## 9 Discussion

This review argues that nutritional adequacy in critical illness should be interpreted contextually rather than only numerically. Guidelines and expert recommendations support early consideration of nutrition, preference for EN when feasible, avoidance of overfeeding, progressive advancement of targets, and continuation of nutrition care beyond the ICU (1, 5, 7, 16, 17). However, these recommendations do not establish one universally valid dose or route for all critically ill patients. Instead, they provide a clinical foundation that must be interpreted according to illness phase, metabolic tolerance, delivery feasibility, and recovery goals.

This phase-specific perspective helps explain why trial findings in critical care nutrition often appear inconsistent. Protein trials and meta-analyses have not shown a uniform benefit of higher protein delivery across broad ICU populations, suggesting that protein effects may depend on timing, phenotype, actual delivered dose, and outcome selection rather than on dose alone (24, 25, 27). Similarly, trials of permissive underfeeding, trophic feeding, and higher energy delivery challenge the assumption that early full caloric replacement is always beneficial, but they should not be interpreted as evidence that prolonged underdelivery is harmless (21, 22, 46). These findings support a staged interpretation: cautious progression during early instability, followed by closer attention to cumulative deficits during stabilization and recovery.

Route and delivery issues further illustrate why prescription alone is insufficient. Feeding intolerance literature shows that inconsistent definitions make it difficult to compare prevalence, consequences, and intervention effects across studies (13–15). Therefore, feeding intolerance should be interpreted not merely as gastrointestinal symptoms, but as a delivery-limiting condition that includes true gastrointestinal dysfunction, avoidable interruptions, aspiration risk, and persistent inability to sustain EN (13–15, 52). Likewise, studies of post-pyloric feeding, volume-based feeding, and protocol optimization suggest that delivery can often be improved, but benefits depend on patient selection, workflow, adherence, and local feasibility (35–37, 52). Thus, precision nutrition requires attention not only to targets but also to whether those targets can be reliably delivered.

The same critical interpretation applies to supplemental PN. CALORIES showed that PN can be a feasible route when EN is not possible, whereas EPaNIC cautioned against premature early supplemental PN during acute illness (57, 58). Meta-analyses suggest that supplemental PN may improve energy and protein delivery when EN is inadequate, but effects on mortality, infection, and length of stay remain inconsistent (58–61). Therefore, PN should not be framed as either routine default or therapeutic failure. It is better understood as a selective, phase-sensitive bridging strategy when EN failure persists, deficits become clinically meaningful, and nutritional risk is high.

A major implication of this review is that critical care nutrition should extend into recovery medicine. Post-ICU literature shows that survivors frequently experience inadequate intake, dysphagia, weakness, muscle loss, and reduced continuity of dietetic care after ICU discharge (16–20, 65). However, much of this evidence remains observational or narrative, and no single validated post-ICU nutrition pathway has been established. Therefore, recovery-phase nutrition should be treated as a structured care priority, but future studies must test specific nutrition-rehabilitation pathways using outcomes such as muscle preservation, mobility, independence, quality of life, and return to daily function (17, 66, 69).

Future research should move beyond broad average treatment effects and ask which patients, in which phase, under which delivery conditions, benefit from which nutrition strategy. This will require clearer phase definitions, better phenotyping, rigorous measurement of actual exposure, and recovery-oriented endpoints. Taken together, current evidence supports a shift from static dose-based thinking toward a longitudinal model of precision nutrition that is phase-specific, tolerance-informed, delivery-aware, and explicitly linked to functional recovery.

## 10 Limitations

This was a structured narrative review, not a systematic review. Study selection was relevance based, screening was not performed independently in duplicate, and no formal risk-of-bias assessment or certainty grading was undertaken. The evidence base is heterogeneous in population, phase definition, intervention dose, and outcome selection. Several recommendations rely on guidelines, physiological rationale, or expert interpretation when RCT evidence is absent or conflicting. The operational phase criteria, escalation points, and follow-up intervals proposed here are an author-derived synthesis and have not been validated as a clinical algorithm. They should therefore support, not replace, patient-specific judgment and local protocols.

The prioritization of recent literature may have underrepresented earlier evidence that was not retained as a landmark study. We did not perform quantitative comparison of competing guidelines or assess whether each component of the framework improves outcomes when implemented together. Resource availability, staffing, access to indirect calorimetry, post-pyloric placement, and post-ICU services will influence feasibility. These constraints limit generalizability and reinforce the need for prospective validation in different health systems.

## 11 Conclusion

Precision nutrition in critical illness is best understood as phase-specific interpretation of existing evidence. Safe initiation during acute instability, progressive correction of persistent deficits during stabilization, preservation of enteral delivery, selective use of supplemental PN, and structured post-ICU follow-up form a single continuum. The framework requires prospective validation, but it provides a transparent basis for matching nutritional strategy to physiology, deliverability, and functional recovery.

Its practical use depends on documenting why the present strategy is appropriate, what evidence supports it, how much nutrition is actually delivered, and which clinical change will prompt reassessment. This discipline may be more important than pursuit of a single universal target because it makes uncertainty, implementation failure, and recovery needs visible throughout the course of care.
